# Capacity building for pediatric neuro-oncology in Pakistan- a project by my child matters program of Foundation S

**DOI:** 10.3389/fonc.2024.1325167

**Published:** 2024-02-29

**Authors:** Naureen Mushtaq, Bilal Mazhar Qureshi, Gohar Javed, Nabeel Ashfaque Sheikh, Saqib Kamran Bakhshi, Altaf Ali Laghari, Syed Ather Enam, Shayan Sirat Maheen Anwar, Kiran Hilal, Arsalan Kabir, Alia Ahmad, Amber Goraya, Anum Salman Mistry, Aqeela Rashid, Ata Ur Rehman Maaz, Muhammad Atif Munawar, Atiq Ahmed Khan, Farrah Bashir, Hina Hashmi, Kamran Saeed, Kumail Khandwala, Lal Rehman, Michael C. Dewan, Muhammad Saghir Khan, Muneeb uddin Karim, Najma Shaheen, Nida Zia, Nuzhat Yasmeen, Raheela Mahmood, Riaz Ahmed Raja Memon, Salman Kirmani, Shahzadi Resham, Shazia Kadri, Shazia Riaz, Syed Ahmer Hamid, Tariq Ghafoor, Uzma Imam, Yaseen Rauf Mushtaq, Zulfiqar Ali Rana, Eric Bouffet, Khurram Minhas

**Affiliations:** ^1^ Peadiatric Oncology Section, Department of Oncology, Aga Khan University, Karachi, Pakistan; ^2^ Radiation Oncology Section, Department of Oncology, Aga Khan University, Karachi, Pakistan; ^3^ Neurosurgery Section, Department of Surgery, Aga Khan University, Karachi, Pakistan; ^4^ Medical Oncology, Shaukat Khanum Memorial Cancer Hospital and Research Centre, Karachi, Pakistan; ^5^ Aga Khan University, Karachi, Pakistan; ^6^ Department of Radiology, Aga Khan University, Karachi, Pakistan; ^7^ Department of Oncology, Aga Khan University, Karachi, Pakistan; ^8^ Department of Pediatric Oncology, Children’s Hospital and Institute of Child Health, Lahore, Pakistan; ^9^ Department of Radiology Children’s Hospital and Institute of Child Health, Lahore, Pakistan; ^10^ Department of Pediatric Oncology, Shaukat Khanum Memorial Cancer Hospital and Research Centre, Lahore, Pakistan; ^11^ Sidra Medicine, Doha, Qatar; ^12^ Department of Radiation Oncology, Northwest General Hospital and Research Center, Peshawar, Pakistan; ^13^ Department of Neurosurgery, Ruth K. M. Pfau Civil Hospital, Karachi, Pakistan; ^14^ Jinnah Postgraduate Medical Centre, Aga Khan University, Karachi, Pakistan; ^15^ Pakistan Institute of Medical Sciences, Islamabad, Pakistan; ^16^ Vanderbilt University Medical Center, Nashville, TN, United States; ^17^ Department of Pediatrics, King Faisal Specialist Hospital and Research Center, Madinah, Saudi Arabia; ^18^ Shaukat Khanum Memorial Cancer Hospital and Research Centre, Lahore, Pakistan; ^19^ Indus Hospital & Health Network, Karachi, Pakistan; ^20^ Atomic Energy Medical Centre, Aga Khan University, Karachi, Pakistan; ^21^ Liaquat University of Medical and Health Sciences, Jamshoro, Pakistan; ^22^ Jinnah Medical College and Teaching Hospital, Peshawar, Pakistan; ^23^ Children’s Hospital and Institute of Child Health, Lahore, Pakistan; ^24^ Combined Military Hospital, Rawalpindi, Pakistan; ^25^ Child Aid Association, National Institute of Child Health, Karachi, Pakistan; ^26^ Neurosurgery Department, Patel Hospital, Karachi, Pakistan; ^27^ Children’s Hospital & The Institute of Child Health Multan, Multan, Pakistan; ^28^ The Hospital for Sick Children (SickKids), Toronto, ON, Canada

**Keywords:** pediatric neuro-oncology, capacity-building, multidisciplinary tumor boards, treatment protocols, fellowship program, low-middle income countries, collaborative initiative

## Abstract

**Introduction:**

Initiated in June 2019, this collaborative effort involved 15 public and private sector hospitals in Pakistan. The primary objective was to enhance the capacity for pediatric neuro-oncology (PNO) care, supported by a My Child Matters/Foundation S grant.

**Methods:**

We aimed to establish and operate Multidisciplinary Tumor Boards (MTBs) on a national scale, covering 76% of the population (185.7 million people). In response to the COVID-19 pandemic, MTBs transitioned to videoconferencing. Fifteen hospitals with essential infrastructure participated, holding monthly sessions addressing diagnostic and treatment challenges. Patient cases were anonymized for confidentiality. Educational initiatives, originally planned as in-person events, shifted to a virtual format, enabling continued implementation and collaboration despite pandemic constraints.

**Results:**

A total of 124 meetings were conducted, addressing 545 cases. To augment knowledge, awareness, and expertise, over 40 longitudinal lectures were organized for healthcare professionals engaged in PNO care. Additionally, two symposia with international collaborators and keynote speakers were also held to raise national awareness. The project achieved significant milestones, including the development of standardized national treatment protocols for low-grade glioma, medulloblastoma, and high-grade glioma. Further protocols are currently under development. Notably, Pakistan's first pediatric neuro-oncology fellowship program was launched, producing two graduates and increasing the number of trained pediatric neuro-oncologists in the country to three.

**Discussion:**

The initiative exemplifies the potential for capacity building in PNO within low-middle income countries. Success is attributed to intra-national twinning programs, emphasizing collaborative efforts. Efforts are underway to establish a national case registry for PNO, ensuring a comprehensive and organized approach to monitoring and managing cases. This collaborative initiative, supported by the My Child Matters/Foundation S grant, showcases the success of capacity building in pediatric neuro-oncology in low-middle income countries. The establishment of treatment protocols, fellowship programs, and regional tumor boards highlights the potential for sustainable improvements in PNO care.

## Introduction

Pediatric neuro-oncology (PNO), a field dedicated to addressing central nervous system cancers in the 0-18 age group, carries immense significance worldwide. Pediatric brain tumors represent the leading cause of cancer related mortality in high income countries (HICs) (1). However, the gravity of the situation is even more pronounced in lower-middle income countries (LMIC) such as Pakistan, due to the scarcity of resources and facilities dedicated to PNO care.

In Pakistan, a country with the fifth-largest population in the world, healthcare resources are severely limited (1, 2). The doctor-to-patient ratio stands at 1.1:1000 and over 58% of the population must pay for healthcare expenses out of their own pocket, rendering treatment for complex diseases such as pediatric neuro-oncology a privilege for many (3, 4).

At the time of initiation of this capacity building effort, there were 13 Pediatric Oncology centers in Pakistan, 22 pediatric oncologists, and one trained pediatric neuro-oncologist. While there were Pediatric Hematology/Oncology fellowship programs in Pakistan, there was no dedicated fellowship for Pediatric Neuro-oncology. Additionally, there are no specialized pediatric neurosurgeons in the country and no hospitals other than AKUH with dedicated Multidisciplinary Teams (MDT) for managing children with brain tumors.

In Pakistan, nearly 39 percent of the population is under the age of 18 and data on PNO cases is scarce. A recent analysis of the 2020 GLOBOCAN approximated that brain tumors were the most prevalent cause of cancer-related mortality in a majority of the Eastern Mediterranean region (including Pakistan), and that brain tumors had an estimated age-standardized incidence rate of 1.3 per 100,000 patients (5). Studies conducted in single-center settings have revealed the prevalence of primary brain tumors among pediatric cancers to be around 20-22 percent (6, 7). Additionally, the overall survival rate for these cases remains low, hovering at approximately 25 percent (8). Contributing factors such as delayed diagnoses, inadequate resources, training and a lack of multidisciplinary coordination among healthcare professionals warrants capacity building measures to improve patient outcomes (9).

The initiation of a twinning program in June 2014 marked a significant milestone in the collaboration between the Hospital for Sick Children (SickKids) in Toronto, Canada, and Aga Khan University Hospital (AKUH) in Pakistan. This collaborative endeavor aimed to establish multidisciplinary tumor boards (MTBs) and conduct in-depth reviews of challenging cases at SickKids, ultimately enhancing patient management and prognostication. The program signifies a dedicated effort to improve the overall quality of care through the sharing of expertise and resources between these two institutions. The program’s achievements became evident through the adoption of refined and individualized management strategies, increased referrals to tertiary healthcare facilities, and the improved diagnostic facilities within the Pakistani healthcare landscape (10). Building upon the success of the SickKids collaboration, the Aga Khan University Hospital (AKUH) embarked on a mission to expand pediatric neuro-oncological care in Pakistan. This initiative was supported by a grant secured under a My Child Matters/Foundation S call in 2018.

## Objectives

The objectives of this initiative were centered on establishing Pediatric Neuro-Oncology (PNO) Multidisciplinary Teams (MDTs), educating medical staff and to develop diagnostic and management guidelines in collaborating children’s hospitals across Pakistan for optimal care of pediatric patients with brain tumors. This work aimed to implement National Pediatric NO Tumor Boards and monthly tumor boards in regional centers to facilitate regular discussions among MDT members for the optimization of treatment strategies. A specific focus was placed on educating clinical staff engaged in the care of children with brain tumors, with the objective of elevating the quality of care at regional levels. The overarching goal was to develop and disseminate national protocols and guidelines for various medical specialties, including Nurses, Oncologists, Histopathologists, Neurosurgeons, Radiation Oncologists, and Radiologists, thereby standardizing and improving patient outcomes. Additionally, the research aimed to enhance diagnostic capabilities in Neuroradiology and Neuropathology in regional centers to ensure accurate and timely assessments of pediatric brain tumors, contributing to a comprehensive and standardized approach to pediatric neuro-oncology care in Pakistan.

The initiative aimed to train a significant number of healthcare practitioners, with projections indicating that approximately 85-100 physicians and 40-60 nurses would receive training through workshops. Additionally, around 80-100 healthcare professionals were expected to benefit from regional tumor boards. Furthermore, a one-year fellowship training position would be initiated for specialized training in pediatric neuro-oncology at the Aga Khan University Hospital (AKUH).

## Methodology

The Multidisciplinary Tumor Boards (MTBs) were established on a national scale, with at least one MDT in each province. Collectively, these MTBs cover 76% of the total population, equivalent to 185.7 million people. With the onset of COVID-19 pandemic, the intercity MDTs transitioned to videoconferencing for their operations. Outside Karachi MTBs had an online videoconferencing format from the beginning.

**Figure 1:**
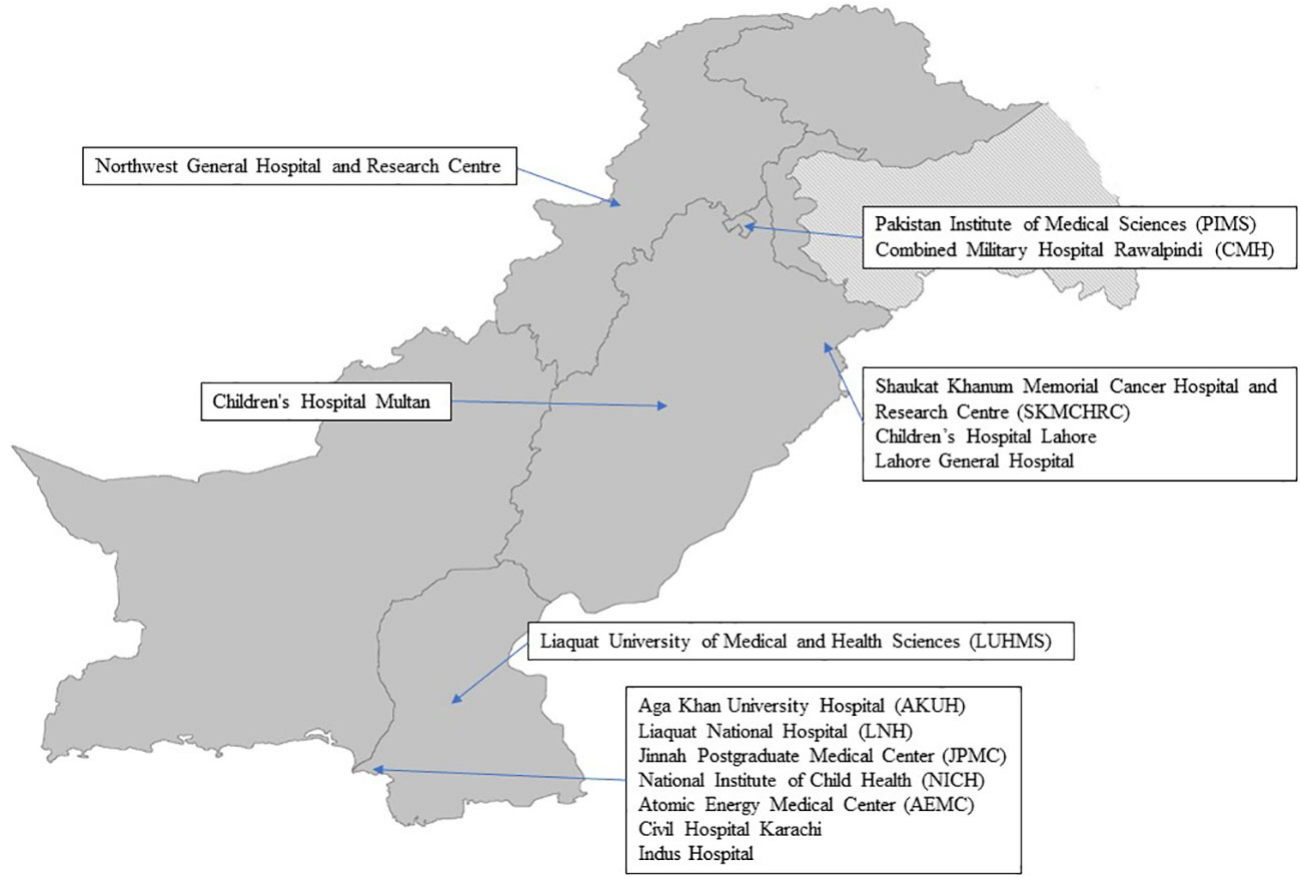
PNO Initiative: Hospitals Participating in Multidisciplinary Tumor Boards.

The participating hospitals, depicted in **Figure 1**—a map showcasing all 15 hospitals—originally began with eight hospitals and later expanded to encompass a total of fifteen hospitals across four provinces. The selection criteria for these hospitals were contingent upon their existing infrastructure, requiring the presence of on-site neurosurgery and neuro-oncology departments, as well as convenient access to radiation oncology services.

The tumor boards were initially planned as monthly sessions, featuring multidisciplinary specialists from each participating institution. These sessions, intended to last approximately 60 to 90 minutes, were structured with the flexibility to increase in frequency based on capacity requirements. Notably, the 15 medical centers actively participated in three major tumor boards: Punjab Tumor Board, JPMC Tumor Boards, and LUMHS Tumor Board, each conducted separately. Centers joined the tumor board closest to them geographically for collaboration. To preserve patient confidentiality, cases were anonymized, referring only to the patient’s age, gender, and diagnosis. These sessions focused on cases presenting diagnostic or treatment-related challenges, with the overarching objective of formulating comprehensive, individualized, and well-coordinated management plans. Pertinent recent medical literature was scrutinized and subsequently shared among participants to inform specific aspects of clinical decision-making. It is essential to note that all cases underwent rigorous peer review within their respective departments, constituting an additional layer in the patient safety-centered quality management process.

National and international awareness and educational initiatives were initially planned as in-person events, including Pediatric Neuro-Oncology (PNO) symposiums and workshops including workshops for nurses. However, due to the COVID-19 pandemic, these events were transitioned to a virtual format. Despite the initial intent for in-person sessions, the adaptation to virtual platforms allowed for continued implementation, including multiple online lectures, thereby ensuring the dissemination of knowledge, and fostering collaboration.

## Results

**Figure 2:**
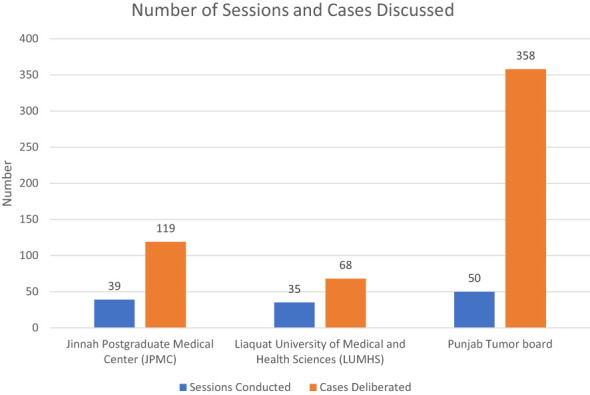
Total Sessions and Discussed Cases in Each Tumor Board.

In total, 124 Multidisciplinary Team (MDT) sessions were conducted from June 19 onwards, persisting to the present day, with the data included up to August ‘23. These sessions comprised 39 meetings at Jinnah Postgraduate Medical Center (JPMC), 35 sessions at Liaquat University of Medical and Health Sciences (LUMHS) tumor boards, and 50 sessions at the Punjab Tumor board (**Figure 2**).

**Figure 3:**
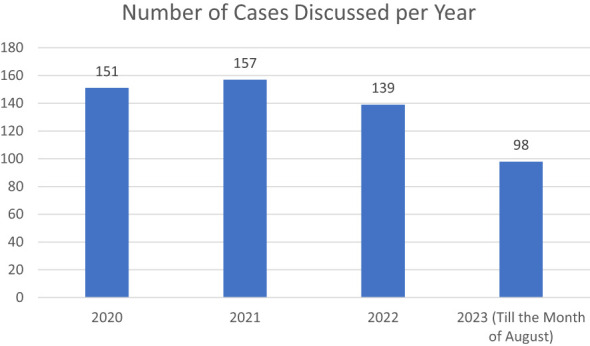
Number of Cases Discussed per year.

The total number of cases deliberated upon amounted to 545, with an average of 4.4 cases discussed per session, spanning a range from 1 to 13 cases. Notably, the majority of cases (66%, n=358) originated from the Punjab Tumor boards, aligning with the higher participation rate of institutions in this particular tumor board. Among the cases discussed, there were 229 female patients and 316 male patients. The mean age of patients discussed was 8.92 years at JPMC, 8.8 years at LUMHS, and 7.37 years at the Punjab Tumor Boards (**Figure 3**).

**Figure 4:**
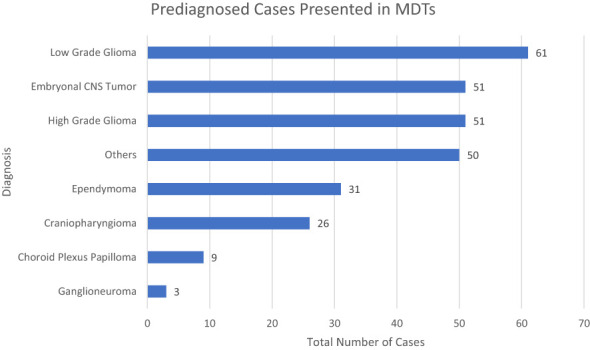
Distribution of Diagnosed Cases in Pediatric Neuro-Oncology (PNO) by Tumor Type.

Of the cases examined, 263 patients underwent discussion before receiving histopathological disease confirmation. Among this subgroup, space-occupying lesions within the posterior fossa (33%) and the supratentorial space (22%) comprised over half of the cases. Among cases (n=282) with histopathological diagnoses confirmed before discussion, there was notable heterogeneity, encompassing 48 different diagnostic categories, as shown in **Figure 4**.

Results of the initiative included the organization of Pediatric Neuro-Oncology (PNO) symposiums aimed at increasing national and international awareness. The inaugural virtual symposium in November 2020 attracted many in the field, bringing together 1126 participants from 58 countries. Themed ‘Working Together for Better Outcomes,’ it highlighted PNO’s significance nationally and globally. Following the inaugural symposium, a second hybrid symposium in November 2021 aimed to establish pediatric neuro-oncology as a vital sub-specialty in lower- and middle-income countries (LMICs). Achievements included 31 international speaker presentations, multiple virtual sessions with 1,007 participants worldwide, and physical sessions with 159 participants (**Table 1**). These sessions covered challenges in pediatric brain tumor care and the need for multidisciplinary collaboration.

**Table 1:** Number of Participants in The Second Hybrid Symposium.

Country	Number of Participants	Country	Number of Participants
Algeria	1	Malaysia	30
Argentina	2	Mexico	14
Armenia	2	Morocco	5
Australia	8	Nepal	1
Bahrain	5	Netherlands	2
Bangladesh	3	New Zealand	1
Bolivia	1	Nigeria	1
Bosnia & Herzegovina	2	Oman	6
Brazil	1	Pakistan	563
Canada	40	Palestinian Territories	1
China	5	Peru	6
Colombia	5	Philippines	6
Croatia	2	Portugal	2
Croatia	1	Puerto Rico	2
Czech Republic	2	Qatar	4
Ecuador	1	Russia	5
Egypt	26	Saudi Arabia	45
Ethiopia	4	Slovakia	2
Germany	5	Slovenia	5
Germany	4	South Africa	12
Ghana	4	Spain	12
Guatemala	2	Tunisia	1
Honduras	2	Uganda	5
Hong Kong	10	Ukraine	11
India	5	United Arab Emirates	10
Indonesia	10	United Kingdom	15
Jordan	25	United States	45
Kuwait	4	Uruguay	2
Lebanon	3	Yemen	1
Libya	7		

As part of the initiative’s outcomes, before the COVID-19 pandemic physical workshops were conducted, engaging a total of 159 participants across four distinct sessions. Commencing in Lahore in 2019 at the Children’s Hospital Lahore, the first workshop facilitated discussions on the foremost challenges associated with accessing and upholding the quality of care for pediatric brain tumor patients in resource-limited environments. Subsequent workshops took place at LUMHS in 2019, and AEMC Karachi in January 2020, both delving into the latest diagnostic, pathological, and genetic advancements to enhance the evaluation of children with brain tumors. Additionally, a dedicated physical nursing workshop was conducted in 2021.

The conversion of physical workshops to an online format also led to the development of a longitudinal lecture series, with 41 lectures attracting over 2500 participants from 17 countries. These lectures, lasting 60-90 minutes each, were delivered by subject matter experts and conducted via video conferencing software. Members were also extended invitations to monthly journal clubs organized and led by fellows at Aga Khan University Hospital (AKUH). These journal club meetings have been held monthly, commencing in 2020 and continuing up to the present date, with an ongoing frequency.

Despite a significant burden of pediatric neuro-oncology (PNO) tumors in Pakistan, the country initially had only one trained and dedicated Pediatric Neuro-oncologist. In response to this gap, a 12-month academic and clinical fellowship program was initiated at the Aga Khan University Hospital (AKUH) in 2020. The program currently offers one fellowship position annually, with plans for expansion based on its success. To date, two fellows have graduated from the fellowship program and are practicing in major cities across the country.

Another outcome facilitated by the grant is the formulation of standardized protocols specifically tailored to address various neuro-oncological tumors. These guidelines cover aspects of patient care, including clinical evaluation, imaging techniques, surgical procedures, chemotherapy regimens, and radiation therapy protocols. Furthermore, the guidelines offer insights into the administration of chemotherapeutic agents, dosage considerations, management of adverse effects, and a framework for post-treatment follow-up.

These guidelines have received official endorsement from both the Pakistan Society of Pediatric Oncology (PSPO) and the Pakistan Society of Neuro-Oncology (PASNO), for neuro-oncological conditions, including medulloblastoma, low-grade glioma, and high-grade glioma. The standardized protocols have been adopted by all 15 participating centers (11).

An additional result that was not planned included the establishment of the Children Brain Tumor Initiative Pakistan (CBTIP): a pediatric neuro-oncological network. This initiative includes the development of an online portal aimed at facilitating case registration and inquiries from patients and healthcare professionals nationwide. The project is considered a unique endeavor in its domain (12).

The dedicated CBTIP website serves as a resource for the early diagnosis and prompt referral of children suspected of or diagnosed with brain tumors. Additionally, a WhatsApp group comprising 90% of pediatric neuro-oncologists (PNO) physicians in Pakistan has been established, with 305 members as of September 2023. This group functions as a platform for knowledge sharing, seeking guidance, and staying updated on institutional developments.

## Discussion

A series of studies have demonstrated the effectiveness of telemedicine and twinning programs in improving the quality of pediatric oncology care in developing countries. Al-Jadiry et al. reported significant improvements in diagnoses and management of pediatric cancer in Iraq through a partnership with Sapienza University of Rome, which included teleconsultations and pathology reviews (13). Similarly, Qaddoumi and Bouffet found that e-mail exchanges enhanced a neuro-oncology twinning program between Jordan and Canada, facilitating communication and collaboration (14). Amayiri further supported the sustainability and impact of video-teleconferencing in pediatric neuro-oncology, emphasizing the role of commitment and motivation in maintaining such initiatives (15). These studies collectively highlight the potential of telemedicine and twinning programs in bridging the gap in pediatric oncology care between developed and developing countries.

In terms of the anticipated impact, it was estimated that the initiative benefited up to 1500 pediatric patients over the course of 3 years. Beyond this direct patient impact, it improved practices of health care professionals involved in PNO care. Moreover, it was anticipated that improved clinical outcomes would influence the perspectives and priorities of governmental health authorities, fostering greater attention to the complex and underserved pediatric population with neuro-oncological conditions.

It is worth noting that a pre-tumor board management plan was not established, rendering quantitative assessment of the impact of Multidisciplinary Tumor Boards (MTBs) on altering management plans unfeasible. Nevertheless, based on the collective experience and testimonies of participants, MTBs emerged as a valuable platform for educating participants, optimizing treatment modalities at each center, and fostering the potential for referrals to institutions for further management of complex cases. All participants expressed their enthusiasm for the monthly tumor board sessions and their intent to continue this practice, showing the accrued benefits derived from this collaborative endeavor.

This initiative spans multiple institutions and referral centers, composed of healthcare providers from diverse institutions, helping to address the complex challenges posed by PNO by fostering multidisciplinary relationships. It allows physicians to communicate, coordinate, and streamline patient care across institutional boundaries. This approach recognized and utilized the capabilities of participating institutions, some specializing in radiotherapy while others in chemotherapy, and leverages these strengths to benefit patients in their own regions.

Furthermore, the Principal Investigator (PI) of the grant has played a pivotal role by keeping open lines of communication for PNO physicians to reach out with inquiries, concerns, and requests for insights 24/7. This accessibility to expert guidance has greatly enhanced the quality of care provided to pediatric neuro-oncology patients.

The advent of the website intends to grow connections between patients and physicians in this field. By swiftly connecting parents of affected children to pediatric neuro-oncology centers equipped with the necessary infrastructure, the website will effectively reduce diagnostic delays and ensures timely intervention by trained specialists, ultimately leading to improved patient outcomes. This innovative platform has the potential to make a significant impact in the field of pediatric neuro-oncology care in Pakistan and beyond, underscoring the transformative potential of collaborative initiatives (12).

Looking forward, there is a clear vision of growth in the field of pediatric neuro-oncology in Pakistan. Despite resource limitations, the goal is to equip each pediatric oncology center with dedicated pediatric neuro-oncologists, neuro-radiologists, neuropathologists, neurosurgeons, and radiation oncologists. This growth not only signifies the increasing recognition of the importance of specialized care but also underscores the commitment to providing the best possible outcomes for PNO patients.

One area that requires focused attention and advancement is the generation of pediatric neuro-oncology-related research from Pakistan. While physicians have made substantial progress in offering individualized management of each patient, there is a pressing need to translate this knowledge into published research data.

In addition to the aforementioned priorities, it is essential to emphasize the establishment and significance of a National Cancer Registry. Presently, Pakistan possesses few hospital-based cancer registries at institutions such as AKU, a city-wide Karachi Cancer Registry, and a provincial Punjab Cancer Registry. However, the development of a comprehensive National Cancer Registry is imperative to accurately capture and consolidate data on pediatric neuro-oncological cases. This unified registry will not only provide a more comprehensive and accurate representation of the landscape but also serve as a crucial tool for informed decision-making and strategic planning in the realm of pediatric brain tumor care and ultimately all cancer care.

Our recommendations encompass the establishment of robust pediatric neuro-oncology services in Pakistan, which entail infrastructural enhancements across various domains such as diagnostic imaging, histopathologic analysis, radiation treatment, oncology services, neurosurgery, and post-treatment rehabilitation. Moreover, fostering collaboration among healthcare providers in these disciplines is crucial to deliver comprehensive multidisciplinary care, guided by locally validated protocols. Central to our approach is the emphasis on the implementation of tumor boards in all cancer hospitals in Pakistan, in alignment with our overarching goal of enhancing pediatric neuro-oncology care in LMICs (10, 16).

By sharing our experiences and successes, we hope to offer valuable insights to not only healthcare professionals and institutions within Pakistan but also to LMICs facing similar challenges in pediatric neuro-oncology care.

Our journey demonstrates the potential to pave the way for improved outcomes in the face of limited resources. We also encourage other institutions within Pakistan to consider joining the cause, contributing their expertise and resources to further strengthen the collaborative effort in addressing the pressing issue of pediatric neuro-oncology care in our region.

In summary, the Foundation S grant has demonstrated considerable success in enhancing the landscape of pediatric neuro-oncology care in Pakistan. It has fostered collaboration, established standardized protocols, and created a supportive network of healthcare professionals. However, there remains an imperative need for more programs of a similar nature to further advance research, standardize care, and ultimately improve outcomes for pediatric neuro-oncology patients in Pakistan and beyond. The data shared above affirms the positive impact of such initiatives and emphasizes the potential for transformative change in healthcare delivery.

## Data availability statement

The raw data supporting the conclusions of this article will be made available by the authors, without undue reservation.

## Author contributions

NM: Conceptualization, Funding acquisition, Visualization, Writing – original draft. BQ: Formal analysis, Funding acquisition, Project administration, Writing – review & editing, Supervision. GJ: Writing – review & editing, Funding acquisition, Project administration, Resources, Conceptualization, Supervision. NS: Formal analysis, Writing – original draft, Writing – review & editing. SB: Formal analysis, Writing – original draft, Validation. AL: Investigation, Methodology, Resources, Writing – review & editing, Conceptualization, Validation. SE: Resources, Visualization, Writing – review & editing, Conceptualization, Validation. SA: Investigation, Methodology, Project administration, Writing – review & editing. KH: Investigation, Project administration, Writing – review & editing. AK: Formal analysis, Writing – original draft. AA: Investigation, Methodology, Resources, Writing – review & editing. AG: Investigation, Resources, Writing – review & editing. AM: Investigation, Methodology, Writing – review & editing. AR: Investigation, Methodology, Resources, Writing – review & editing. AM: Investigation, Supervision, Writing – review & editing. MM: Investigation, Writing – review & editing. AK: Investigation, Resources, Writing – review & editing. FB: Data curation, Investigation, Project administration, Writing – review & editing. HH: Investigation, Writing – review & editing. KS: Investigation, Writing – review & editing. KK: Investigation, Project administration, Writing – review & editing. LR: Investigation, Methodology, Resources, Writing – review & editing. MD: Data curation, Investigation, Writing – review & editing. MS: Investigation, Writing – review & editing. MK: Investigation, Writing – review & editing. NS: Investigation, Methodology, Writing – review & editing. NZ: Investigation, Resources, Writing – review & editing. NY: Investigation, Resources, Writing – review & editing. RM: Investigation, Resources, Writing – review & editing. RM: Investigation, Methodology, Writing – review & editing. SK: Investigation, Methodology, Writing – review & editing. SR: Investigation, Methodology, Writing – review & editing. SK: Investigation, Methodology, Writing – review & editing. SR: Investigation, Methodology, Resources, Writing – review & editing. SH: Investigation, Project administration, Writing – review & editing. TG: Investigation, Resources, Writing – review & editing. UI: Investigation, Methodology, Resources, Writing – review & editing. YM: Investigation, Writing – review & editing. ZR: Investigation, Writing – review & editing. EB: Funding acquisition, Supervision, Writing – review & editing. KM: Conceptualization, Formal analysis, Funding acquisition, Resources, Writing – original draft, Writing – review & editing.
